# Consistent adherence to biologic therapies is critical for improving outcomes in asthma

**DOI:** 10.1016/j.jacig.2026.100771

**Published:** 2026-08-07

**Authors:** Njira L. Lugogo, Brian Modena, Justin Kwiatek, Jiaxuan Wang, Peter Howarth, Riyad Al-Lehebi, Anna Vichiendilokkul, Urvee Karsanji, Jeremiah Hwee, Gerald Smith, Riley Geason, Arijita Deb, Rafael Alfonso-Cristancho

**Affiliations:** a Division of Pulmonary and Critical Care Medicine, University of Michigan, Ann Arbor, Mich; b Modena Health, La Jolla, Calif; c Medical Affairs, GSK, Upper Providence, Pa; d Cytel, Inc, Vancouver, British Columbia, Canada; e Global Medical Affairs, Specialty Care, GSK, London, United Kingdom; f Department of Pulmonology, King Fahad Medical City, Riyadh, Saudi Arabia; g College of Medicine, Alfaisal University, Riyadh, Saudi Arabia; h Global Medical Affairs, GSK, Collegeville, Pa; i Real World Biostatistics, GSK, London, United Kingdom; j Global Epidemiology, GSK, Mississauga, Ontario, Canada; k Real World Evidence, Cytel Inc, Boston, Mass; l Global Real-World Evidence & Health Outcomes Research, GSK, Collegeville, Pa

**Keywords:** Adherence, asthma, biologics, cost, exacerbations, health care resource utilization, group-based trajectory modeling

## Abstract

**Background:**

Evidence on real-world adherence to asthma biologic therapies and the relevance to clinical outcomes in asthma is limited.

**Objective:**

We sought to evaluate biologic use patterns and the relationships between adherence, clinical, and health care resource utilization (HCRU) outcomes and economic burden in patients with asthma.

**Methods:**

This was a retrospective observational study using Optum’s de-identified Market Clarity Data (Optum Market Clarity) from 2007 to 2023 in adults in the United States with an asthma diagnosis in the 12 months before the first biologic dose (index date) who had ≥12 months of follow-up. Medication possession ratio (MPR; number of doses taken/dosing intervals) and group-based trajectory modeling (GBTM) identified distinct patient adherence patterns and assessed impact on exacerbations, HCRU, and costs (all-cause/asthma-related).

**Results:**

Overall, 10,088 eligible patients were included in the MPR analysis; 54.7% discontinued treatment, 5.2% were minimally adherent, 20.3% were partially adherent, and 19.8% were adherent. Of these, 9,553 did not switch biologic during follow-up and were included in the GBTM analysis. For patients with 1 or more follow-up doses (n = 8,151), MPRs revealed suboptimal biologic adherence (average range, 40.6%-64.0%). GBTM identified 7 adherence clusters, with distinct adherence trajectories categorized from highest to lowest levels of biologic adherence. Patients with the highest adherence generally demonstrated the best outcomes. In patients in less adherent groups, increases were seen in exacerbations defined by asthma-related hospitalizations, all-cause HCRU, and all-cause health care costs. HCRU and asthma-related costs were generally lower for patients with the highest adherence.

**Conclusions:**

Treatment adherence was consistently associated with better all-cause and asthma-related outcomes, highlighting the importance of biologic adherence in patients with asthma.

## Graphical abstract

**Figure:**
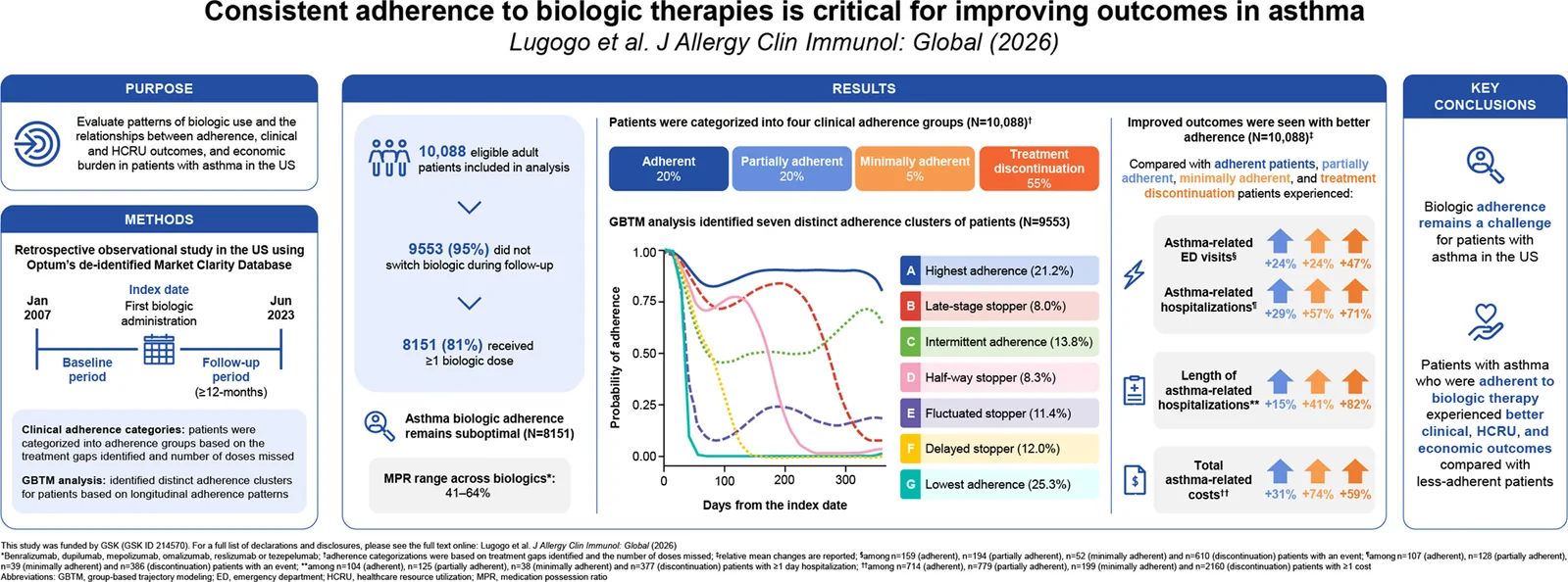

The overarching goal of severe asthma management is to prevent asthma-related deaths and reduce the substantial burden the disease places on individuals, their families, communities, and health care systems.^1^ Patients with severe asthma typically require maintenance therapy with medium- or high-dose inhaled corticosteroids (ICSs) in combination with a second controller to achieve disease control, with maintenance oral corticosteroids (OCSs) used as a last resort.^1^ If their condition remains uncontrolled, the Global INitiative for Asthma guidelines recommend the addition of biologic therapy to standard-of-care treatment.^1^ Currently, there are 6 biologic therapies approved for the treatment of asthma^2^: benralizumab (anti–IL-5R), dupilumab (anti–IL-4Rα), mepolizumab (anti–IL-5), omalizumab (anti-IgE), reslizumab (anti–IL-5), and tezepelumab (anti–thymic stromal lymphopoietin).^3^, ^4^, ^5^, ^6^, ^7^, ^8^ Treatment with biologics has been shown to reduce exacerbations and hospitalizations; improve lung function, asthma control, and health-related quality of life; and limit the use of OCSs.^9^

Despite these advancements in asthma treatment options, poor adherence to prescribed therapies remains a significant challenge in some patients.^10^ There could be multiple reasons for poor adherence in patients with asthma, including forgetfulness, complex inhaler schedules/techniques, patient education, socioeconomic barriers, and limited access to health care resources.^10^^,^^11^ Regardless of the cause, suboptimal asthma treatment adherence may lead to inconsistent control of underlying chronic inflammation and is associated with poor clinical outcomes, increased risk of exacerbations, and, subsequently, increased health care resource utilization (HCRU).^12^, ^13^, ^14^, ^15^ Nonadherence to biologic therapies has been associated with similar negative outcomes / increased HCRU and health care costs in various diseases, including multiple sclerosis^16^ and rheumatoid arthritis.^17^^,^^18^ However, few studies report nonadherence to biologic therapies for asthma and even fewer describe the impact of low adherence on clinically meaningful outcomes.^19^, ^20^, ^21^ There is also considerable variability in the definitions of adherence used in different studies, further complicating the challenge of accurate assessment.^22^

This study assessed patterns of biologic use and their impact on clinical and economic outcomes in patients with severe asthma. Three different methods were used to evaluate adherence, including medication possession ratio (MPR), predefined adherence criteria, and group-based trajectory modeling (GBTM), an innovative method that incorporates the timing and frequency of missed doses to identify distinct adherence patterns beyond static metrics such as MPR. These approaches complement one another and provide a more comprehensive understanding of biologic treatment adherence and its impact on outcomes.

## Methods

### Study design and sample selection

**Fig 1:**
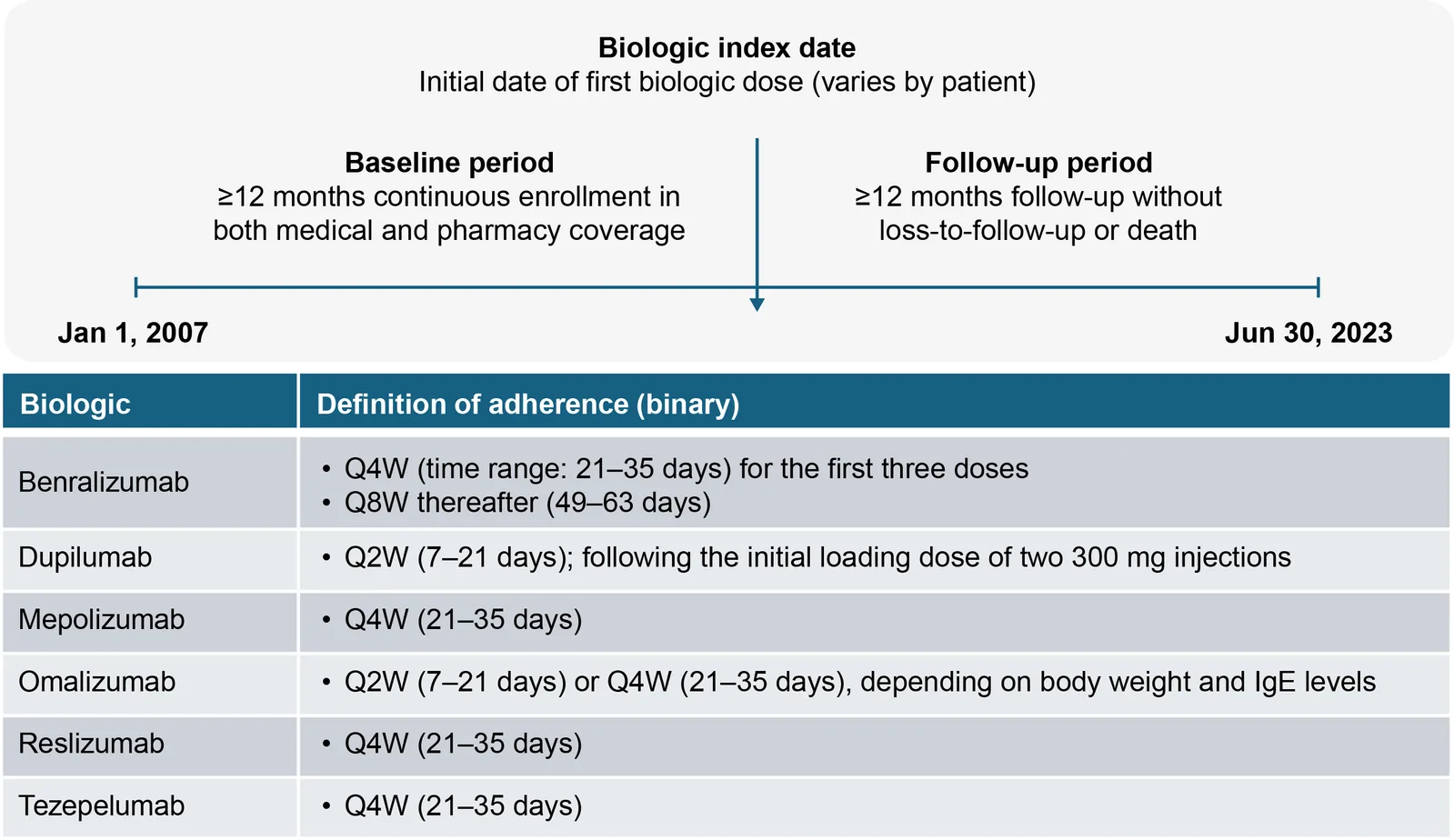
Study design. *Q2W/Q4W/Q8W*, Every 2/4/8 weeks.

This was a retrospective observational study (GSK ID: 214570) of patients with asthma treated with 1 of 6 approved biologics (benralizumab, dupilumab, mepolizumab, omalizumab, reslizumab, and tezepelumab) from January 1, 2007, to June 30, 2023, using Optum’s US de-identified Market Clarity Data (Optum Market Clarity) (Fig 1). Optum Market Clarity is an integrated, multisource data set of medical claims, pharmacy claims, and electronic health records (EHRs). It links EHR data—including laboratory results, vital signs and measurements, diagnoses, procedures, and information derived from unstructured clinical notes using natural language processing—with historical and linked administrative claims data, including pharmacy claims, physician claims, clinical information facility claims, and medications prescribed and administered. Optum Market Clarity is statistically de-identified under the Health Insurance Portability and Accountability Act Privacy Rule’s Expert Determination method and managed according to Optum customer data use agreements.

The index date was defined as the initial date of the first biologic dose administration. The baseline period was defined as the 12 months before the index date (exclusive), and the follow-up period was the 12 months after the index date (inclusive).

### Patients

Eligible patients were 18 years or older on the index date and had a diagnosis of asthma during the baseline period (12 months before the first biologic dose), identified using the *International Classification of Diseases, Ninth or Tenth Revision* codes (*ICD-9-CM* 493∗ or *ICD-10-CM* J45∗) from either EHRs or the claims database. Patients must have had initiated treatment with one of the specified approved asthma biologics as identified through prescriptions, administrations, procedures, and medical services from Healthcare Common Procedure Coding System and National Drug Codes. Additional inclusion criteria included 12 months or more of clinical activity or continuous enrollment (inclusive of medical and pharmacy benefits) before the index date and 12 months or more of follow-up after the index date (without loss to follow-up or death). To minimize chance of previous biologic experience affecting adherence in this study, patients must have had no previous use of any asthma biologic therapy at any time before the index date. Additional inclusion and exclusion criteria are provided in this article’s Methods section in the Online Repository at www.jaci-global.org.

### Feasibility assessment

Adherence to each biologic was determined over the 12-month follow-up period using the MPR and visualized via heatmaps. Adherence was determined on the basis of prescription dates and approved dosing schedules: benralizumab every 4 weeks for the first 3 doses and then every 8 weeks; dupilumab every 2 weeks; mepolizumab, reslizumab, and tezepelumab every 4 weeks; and omalizumab every 2 or 4 weeks depending on body weight and IgE level. A dynamic ±7-day buffer around the expected treatment dosing date was used to determine the binary adherence indicator (adherent or nonadherent), ensuring maximum capture of adherence data (Fig 1; see also this article’s Methods section in the Online Repository). The schedule for the next dose was then dynamically reset on the basis of the date of the previous dose. The MPR for each biologic was calculated as the total number of days with medication possession divided by the total number of days in the 12-month follow-up period. Although MPR is an established threshold-based metric in adherence research, it is a static metric and not sensitive to differences in patterns of use. Therefore, GBTM was applied to longitudinal binary treatment adherence during the 12-month follow-up period to identify distinct clusters of patients exhibiting similar adherence patterns to provide a dynamic view of long-term adherence patterns and their impact on patient outcomes. Patients were also clinically categorized by level of adherence to each biologic into treatment adherence groups (adherent, partially adherent, minimally adherent, and treatment discontinuation) on the basis of the treatment gaps identified and the number of doses missed (as defined in Table E1 in this article’s Online Repository at www.jaci-global.org). Although traditional adherence categories offer straightforward comparability across groups, GBTM clusters can provide a novel, dynamic, and data-driven understanding of the longitudinal patterns of adherence. This dual approach enables a more comprehensive understanding of adherence behaviors and the impact on patient outcomes.

### Clinical outcomes

The primary clinical outcome was the frequency and proportion of patients experiencing 1 or more exacerbations during the follow-up period across various treatment adherence clusters. Exacerbations were classified as hospitalization-related (defined by asthma-related hospitalizations), emergency department (ED)-related (defined by asthma-related ED visits), or corticosteroid-related (defined by bursts and nonchronic OCS or ICS use) exacerbations. Other outcomes included short-acting β_2_-agonist (SABA) use, frequency and proportion of patients with HCRU, and event-specific health care costs (reported in US dollars) for all-cause and asthma-related events across treatment adherence categories and clusters during the follow-up period. This study also examined patterns of exacerbations before/after the first wave of the coronavirus disease 2019 (COVID-19) pandemic. Further details on clinical outcomes and COVID analyses are provided in this article’s Methods section in the Online Repository.

### Statistical analysis

Descriptive statistical analyses were used to describe patient baseline characteristics. Means (SDs) and medians (interquartile range) were calculated for continuous variables, whereas categorical variables were presented as the number and proportion of patients; standardized mean differences (SMDs) were used to compare patient baseline characteristics across the 6 biologics. All statistical analyses were conducted using 2-sided tests, with statistical significance defined at a *P* value less than .05. Full statistical details (including those for descriptive analyses, heatmaps, longitudinal adherence clusters, and binary adherence over time) are provided in this article’s Methods section in the Online Repository.

### Ethical approval and patient consent

Because this study used routinely collected anonymized data from Optum Market Clarity (ie, no direct patient contact or new data collection), informed consent, ethics committee, or institutional review board approvals were not required, in line with Optum’s guidance. The study complied with all applicable laws, regulations, and guidance regarding patient protection (including patient privacy), all data were de-identified, and all study reports contained only aggregate results.

## Results

### Patient population

In total, 10,088 patients met the eligibility criteria and were included in the MPR feasibility assessment. Of these, 1,301 (12.9%) were prescribed benralizumab, 2,096 (20.8%) dupilumab, 1,545 (15.3%) mepolizumab, 5,048 (50.0%) omalizumab, 84 (0.8%) reslizumab, and 14 (0.1%) tezepelumab. Baseline patient characteristics were broadly similar across all biologics, with only slight to moderate differences observed except for the index year (see Table E2 in this article’s Online Repository at www.jaci-global.org), reflecting the different approval and launch dates for each asthma biologic in the United States. Of the 10,088 eligible patients, 9,553 remained on the same biologic during the 12-month follow-up period and were included in the GBTM analysis. Of these, 1,160 (12.1%) were prescribed benralizumab, 2,008 (21.0%) dupilumab, 1,407 (14.7%) mepolizumab, 4,892 (51.2%) omalizumab, 74 (0.8%) reslizumab, and 12 (0.1%) tezepelumab.

### Treatment adherence MPR analysis

When adherence was assessed among patients who had received 1 or more doses of biologic during follow-up (n = 8,151), the average MPR was 40.6% for tezepelumab, 47.7% for dupilumab, 50.4% for omalizumab, 51.0% for mepolizumab, 53.5% for benralizumab, and 64.0% for reslizumab (see Fig E1 in this article’s Online Repository at www.jaci-global.org). Average MPR was higher (by ∼7%-12%) for all biologics in patients who had received 1 or more doses of biologic compared with the overall population, suggesting better adherence in this subgroup. Treatment adherence patterns visualized using heatmaps showed that all biologic treatments were frequently interrupted and restarted, resulting in many treatment gaps, especially when treatments occurred outside the scheduled timeline (see Fig E2 in this article’s Online Repository at www.jaci-global.org).

### GBTM analysis of adherence clusters

**Fig 2:**
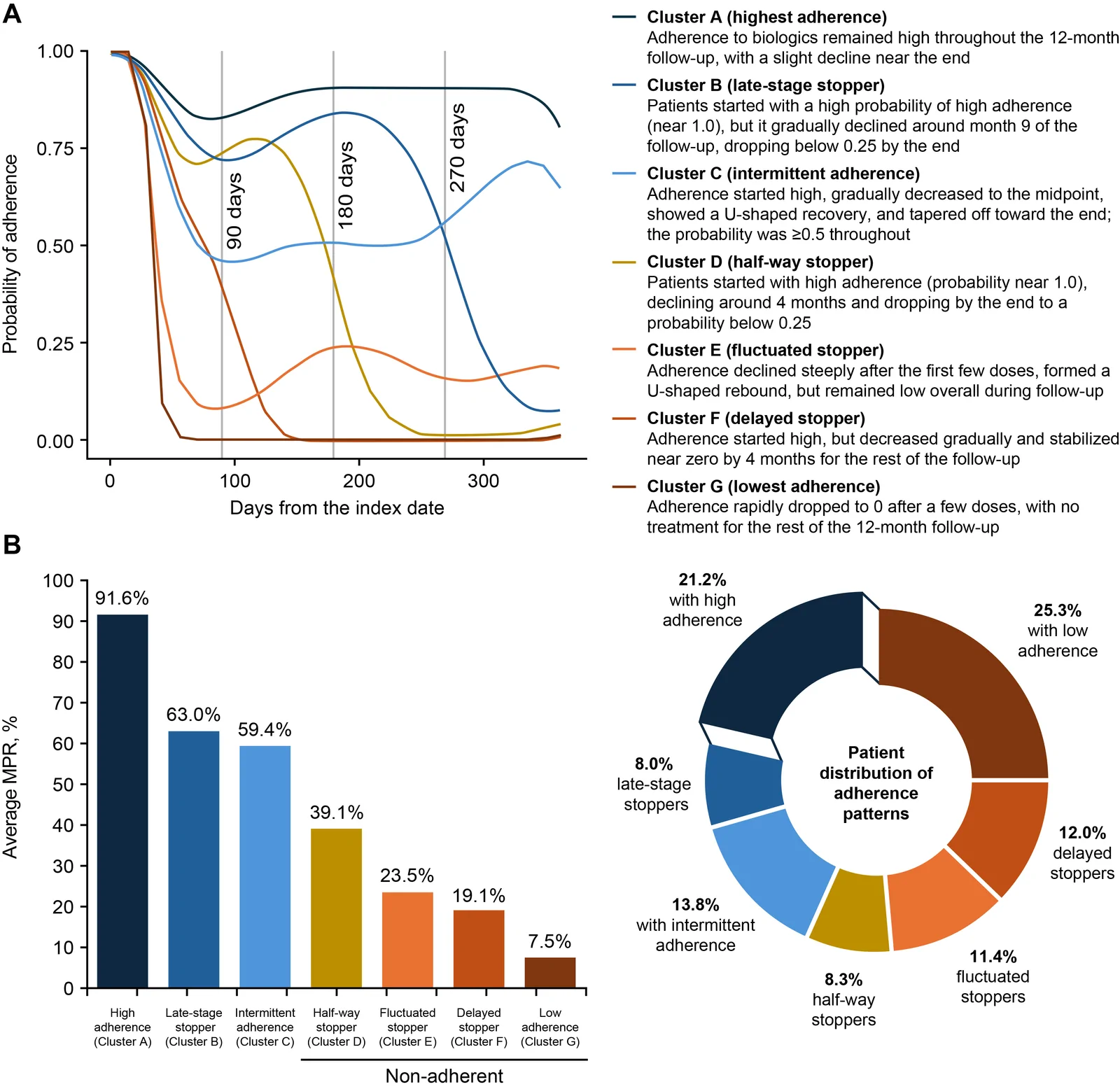
**A** and **B,** Adherence clusters identified using GBTM (Fig 2, *A*) and their average MPR and patient distribution (Fig 2, *B*) in patients who did not switch biologics during the 12-month follow-up period (N = 9,553).

The GBTM analysis identified 7 adherence clusters that demonstrated distinct adherence trajectories over the 12-month follow-up period, revealing consistent, early-drop, initial-only, and U-shaped adherence patterns: cluster A, highest adherence (n = 2,024); cluster B, late-stage stoppers (n = 769); cluster C, intermittent adherence (n = 1,315); cluster D, half-way stoppers (n = 791); cluster E, fluctuation stoppers (n = 1,090); cluster F, delayed stoppers (n = 1,144); and cluster G, lowest adherence (n = 2,420) (Fig 2, *A*). Overall, 21.2% of patients demonstrated the highest adherence (ie, cluster A) achieving an average MPR of 91.6% during the 12-month follow-up, whereas the remaining 78.8% showed various patterns on nonadherence, with more than half having an MPR less than 50% (63.0% for cluster B, 59.4% for cluster C, 39.1% for cluster D, 23.5% for cluster E, 19.1% for cluster F, and 7.5% for cluster G; see Fig 2, *B*).

Baseline patient characteristics across the 7 adherence clusters generally showed slight to moderate imbalances, with SMDs ranging from 0.05 to 0.27 for most parameters (see Table E3 in this article’s Online Repository at www.jaci-global.org). Patients in lower adherence clusters (E-G) tended to have a higher proportion of former smokers, higher body mass index, and higher Charlson comorbidity index scores compared with those in the higher adherence clusters (A-D). The racial composition varied moderately, with White patients comprising 69.9% to 76.2% across clusters, whereas other racial groups varied moderately (SMD, 0.109). Biomarker levels varied moderately across clusters, with median blood eosinophil count ranging from 200.00 to 220.00 cells/μL (SMD, 0.064) and median IgE ranging from 153.50 to 186.05 IU/mL (SMD, 0.066).

### Impact of adherence on outcomes

#### Exacerbations

Among the 10,088 patients included in the treatment adherence categorization analysis, 19.8% were clinically categorized as adherent, 20.3% as partially adherent, 5.2% as minimally adherent, and 54.7% discontinued treatment (defined in Table E1). Adherent patients consistently demonstrated more favorable outcomes across all exacerbation measures compared with those in the partially adherent or nonadherence groups (Table I). Compared with the adherent group, asthma-related hospitalization- and ED visit-related exacerbation rates increased by 23.5% to 71.4% and corticosteroid-related exacerbation rates increased by 6.0% to 12.9% in the less adherent groups (partially/minimally/discontinued treatment). SABA-related exacerbation rates varied slightly in less adherent groups versus the adherent group, but no clear pattern was evident (Table I). Asthma-related ED visits varied across clusters, with the lowest proportion in cluster A (8.3% of patients) and the highest in cluster F (12.4% of patients). Mean ± SD ED visits per patient ranged from 0.16 ± 0.85 in cluster A to 0.29 ± 1.09 in cluster D (81.3% increase; Table II). Asthma-related hospitalizations showed less variability, ranging from 5.1% of patients in cluster A to 8.2% in cluster D. Mean ± SD asthma-related hospitalizations per patient were the lowest in cluster A (0.07 ± 0.36) and the highest in cluster D (0.16 ± 0.69) (128.6% increase; Table II). Corticosteroid use varied modestly across clusters and was the highest in cluster C (37.3% of patients) and the lowest in cluster E (32.9% of patients), with mean events per patient ranging from 1.13 in cluster A to 1.35 in cluster D. SABA use was less affected by adherence patterns, ranging from 76.6% to 80.6% of patients across the clusters. Mean SABA use per patient ranged from 5.27 in cluster A to 6.23 in cluster D (Table II).

**Table I:** Summary of exacerbations by asthma biologic treatment adherence categories during the 12-month follow-up period (N = 10,088)

Clinical outcomes	Adherent (n = 2,000)	Partially adherent (n = 2,045)	Minimally adherent (n = 528)	Treatment discontinuation (n = 5,515)
**Asthma-related ED visits**				
Patients, n (%)	159 (8.0)	194 (9.5)	52 (9.8)	610 (11.1)
Events per patient, mean ± SD	0.17 ± 1.04	0.21 ± 1.02	0.21 ± 0.98	0.25 ± 1.04
Relative mean change∗**(%)**	Reference	+23.5	+23.5	+47.1
Odds of ≥1 event in adherent patients vs other categories, OR (*P* value)	Reference	1.370 (<.001)		
**Asthma-related hospitalizations**				
Patients, n (%)	107 (5.4)	128 (6.3)	39 (7.4)	386 (7.0)
Events per patient, mean ± SD	0.07 ± 0.32	0.09 ± 0.42	0.11 ± 0.50	0.12 ± 0.62
Relative mean change∗**(%)**	Reference	+28.6	+57.1	+71.4
Odds of ≥1 event in adherent patients vs other categories, OR (*P* value)	Reference	1.298 (.016)		
**Corticosteroid use**†				
Patients, n (%)	718 (35.9)	746 (36.5)	196 (37.1)	1,895 (34.4)
Events per patient, mean ± SD	1.16 ± 2.81	1.23 ± 2.68	1.31 ± 2.75	1.26 ± 3.05
Relative mean change∗ (%)	Reference	+6.0	+12.9	+8.6
Odds of ≥1 event in adherent patients vs other categories, OR (*P* value)	Reference	0.965 (.490)		
**SABA use**				
Patients, n (%)	1,577 (78.9)	1,610 (78.7)	419 (79.4)	4,314 (78.2)
Events per patient, mean ± SD	5.42 ± 7.46	5.50 ± 7.20	5.30 ± 6.94	5.61 ± 8.98
Relative mean change∗ (%)	Reference	+1.5	−2.2	+3.5
Odds of ≥1 event in adherent patients vs other categories, OR (*P* value)	Reference	0.975 (.679)		

*OR*, Odds ratio.

∗ Versus the adherent category.

† Corticosteroid use was defined as the use of burst and nonchronic oral or intravenous corticosteroids.

**Table II:** Summary of exacerbations across 7 GBTM clusters among patients who remained on the same biologic during the 12-month follow-up period (N = 9,553)

Clinical outcomes	Cluster A (highest adherent) (n = 2,024)	Cluster B (late-stage stopper) (n = 769)	Cluster C (intermittent adherence) (n = 1,315)	Cluster D (half-way stopper) (n = 791)	Cluster E (fluctuation stopper) (n = 1,090)	Cluster F (delayed stopper) (n = 1,144)	Cluster G (lowest adherent (n = 2,420)
**Asthma-related ED visits**							
Patients with ≥1 event, n (%)	167 (8.3)	75 (9.8)	109 (8.3)	94 (11.9)	99 (9.1)	142 (12.4)	246 (10.2)
Total events per patient during follow-up, mean ± SD	0.16 ± 0.85	0.20 ± 0.88	0.17 ± 0.79	0.29 ± 1.09	0.23 ± 1.12	0.24 ± 0.80	0.23 ± 1.07
Relative mean change vs cluster A (%)	Reference	+25.0	+6.3	+81.3	+43.8	+50.0	+43.8
Odds of ≥1 event in cluster A patients vs other clusters, OR (*P* value)	Reference	1.258 (.010)					
**Asthma-related hospitalizations**							
Patients with ≥1 event, n (%)	104 (5.1)	49 (6.4)	83 (6.3)	65 (8.2)	79 (7.2)	93 (8.1)	143 (5.9)
Total events per patient during follow-up, mean ± SD	0.07 ± 0.36	0.08 ± 0.34	0.09 ± 0.39	0.16 ± 0.69	0.11 ± 0.73	0.12 ± 0.53	0.09 ± 0.44
Relative mean change vs cluster A (%)	Reference	+14.3	+28.6	+128.6	+57.1	+71.4	+28.6
Odds of ≥1 event in cluster A patients vs other clusters, OR (*P* value)	Reference	1.347 (.007)					
**Corticosteroid use**∗							
Patients with ≥1 event, n (%)	709 (35.0)	274 (35.6)	491 (37.3)	288 (36.4)	359 (32.9)	386 (33.7)	802 (33.1)
Total events per patient during follow-up, mean ± SD	1.13 ± 2.67	1.20 ± 2.46	1.19 ± 2.42	1.35 ± 3.28	1.18 ± 2.71	1.25 ± 3.21	1.19 ± 2.90
Relative mean change vs cluster A (%)	Reference	+6.2	+5.3	+19.5	+4.4	+10.6	+5.3
Odds of ≥1 event in cluster A patients vs other clusters, OR (*P* value)	Reference	0.978 (.677)					
**SABA use**							
Patients with ≥1 event, n (%)	1,551 (76.6)	593 (77.1)	1,060 (80.6)	619 (78.3)	864 (79.3)	913 (79.8)	1,874 (77.4)
Total events per patient during follow-up, mean ± SD	5.27 ± 7.47	5.54 ± 7.28	5.31 ± 6.61	6.23 ± 15.04	5.34 ± 6.93	5.41 ± 7.02	5.33 ± 7.70
Relative mean change vs cluster A (%)	Reference	+5.1	+0.8	+16.3	+1.3	+2.7	+1.1
Odds of ≥1 event in cluster A patients vs other categories, OR (*P* value)	Reference	1.125 (.050)					

*OR*, Odds ratio.

∗ Corticosteroid use was defined as the use of burst and nonchronic oral or intravenous corticosteroids.

#### Health care resource utilization

The proportion of patients with all-cause and asthma-related hospitalizations and ED visits was the lowest in the adherent group and increased progressively with declining adherence (Table III). Length of hospitalizations, both all-cause and asthma-related, also increased with declining adherence (Table III). The analysis across the 7 adherence clusters showed a similar trend. Cluster A (highest adherence) consistently showed the lowest HCRU across most all-cause and asthma-related outcomes, whereas cluster D (half-way stoppers), cluster F (delayed stoppers), and cluster G (lowest adherence) had the highest HCRU (Table IV).

**Table III:** Summary of all-cause and asthma-related HCRU by asthma biologic treatment adherence categories during the 12-month follow-up period (N = 10,088)

Clinical outcomes	Adherent (n = 2,000)	Partially adherent (n = 2,045)	Minimally adherent (n = 528)	Treatment discontinuation (n = 5,515)
**All-cause hospitalizations**				
Patients, n (%)	688 (34.4)	771 (37.7)	221 (41.9)	2,525 (45.8)
Events per patient, mean ± SD	1.16 ± 3.47	1.35 ± 3.65	1.49 ± 3.37	1.86 ± 4.47
Relative mean change∗ (%)	Reference	+16.4	+28.4	+60.3
Odds of ≥1 event in adherent patients vs other categories, OR (*P* value)	Reference	1.467 (<.001)		
**Length of all-cause hospitalizations**				
Patients with ≥1 d of hospitalization, n (%)	244 (12.2)	290 (14.2)	89 (16.9)	988 (17.9)
Days per patient, mean ± SD	1.05 ± 5.45	1.27 ± 6.05	1.62 ± 8.40	2.26 ± 9.95
Relative mean change∗ (%)	Reference	+21.0	+54.3	+115.2
Average difference between adherent patients and other categories (*P* value)	Reference	0.916 (<.001)		
**All-cause ED visits**				
Patients, n (%)	591 (29.6)	671 (32.8)	199 (37.7)	2,237 (40.6)
Events per patient, mean ± SD	0.88 ± 2.73	1.01 ± 2.77	1.07 ± 2.49	1.36 ± 3.14
Relative mean change∗ (%)	Reference	+14.8	+21.6	+54.6
Odds of ≥1 event in adherent patients vs other categories, OR (*P* value)	Reference	1.487 (<.001)		
**Asthma-related hospitalizations**				
Patients, n (%)	233 (11.7)	283 (13.8)	79 (15.0)	851 (15.4)
Events per patient, mean ± SD	0.22 ± 1.10	0.28 ± 1.11	0.29 ± 1.09	0.34 ± 1.19
Relative mean change∗ (%)	Reference	+27.3	+31.8	+54.5
Odds of ≥1 event in adherent patients vs other categories, OR (*P* value)	Reference	1.338 (<.001)		
**Length of asthma-related hospitalizations**				
Patients with ≥1 d of hospitalization, n (%)	104 (5.2)	125 (6.1)	38 (7.2)	377 (6.8)
Days per patient, mean ± SD	0.27 ± 1.68	0.31 ± 1.74	0.38 ± 1.91	0.49 ± 3.91
Relative mean change∗ (%)	Reference	+14.8	+40.7	+81.5
Average difference between adherent patients and other categories (*P* value)	Reference	0.176 (.013)		
**Asthma-related ED visits**				
Patients, n (%)	159 (8.0)	194 (9.5)	52 (9.8)	610 (11.1)
Events per patient, mean ± SD	0.17 ± 1.04	0.21 ± 1.02	0.21 ± 0.98	0.25 ± 1.04
Relative mean change∗ (%)	Reference	+23.5	+23.5	+47.1
Odds of ≥1 event in adherent patients vs other categories, OR (*P* value)	Reference	1.370 (<.001)		

*OR*, Odds ratio.

∗ Versus the adherent category.

**Table IV:** Summary of all-cause and asthma-related HCRU across 7 GBTM clusters among patients who remained on the same biologic during the 12-month follow-up period (N = 9,553)

Clinical outcomes	Cluster A (highest adherent) (n = 2,024)	Cluster B (late-stage stopper) (n = 769)	Cluster C (intermittent adherence) (n = 1,315)	Cluster D (half-way stopper) (n = 791)	Cluster E (fluctuation stopper) (n = 1,090)	Cluster F (delayed stopper) (n = 1,144)	Cluster G (lowest adherent) (n = 2,420)
**All-cause hospitalizations**							
Patients with ≥1 event, n (%)	690 (34.1)	282 (36.7)	487 (37.0)	360 (45.5)	467 (42.8)	529 (46.2)	1,139 (47.1)
Total number of events per patient during follow-up, mean ± SD	1.12 ± 3.29	1.34 ± 2.98	1.33 ± 3.05	2.15 ± 5.04	1.69 ± 3.82	1.85 ± 4.65	1.88 ± 4.63
Relative mean change vs cluster A (%)	Reference	+19.6	+18.8	+92.0	+50.9	+65.2	+67.9
Odds of ≥1 event in cluster A patients vs other clusters, OR (*P* value)	Reference	1.480 (<.001)					
**Length of all-cause hospitalizations**							
Patients with ≥1 event, n (%)	236 (11.7)	108 (14.0)	187 (14.2)	143 (18.1)	197 (18.1)	204 (17.8)	441 (18.2)
Total number of days per patient during follow-up, mean ± SD	0.98 ± 5.53	1.33 ± 5.73	1.15 ± 5.31	2.37 ± 10.81	2.29 ± 9.49	2.26 ± 9.93	2.22 ± 9.77
Relative mean change vs cluster A (%)	Reference	+35.7	+17.3	+141.8	+133.7	+130.6	+126.5
Average difference between cluster A patients and other clusters (*P* value)	Reference	0.992 (<.001)					
**All-cause ED visits**							
Patients with ≥1 event, n (%)	591 (29.2)	252 (32.8)	426 (32.4)	321 (40.6)	401 (36.8)	464 (40.6)	1,022 (42.2)
Total number of events per patient during follow-up, mean ± SD	0.85 ± 2.67	1.05 ± 2.49	0.93 ± 2.48	1.63 ± 4.11	1.11 ± 2.57	1.34 ± 3.21	1.37 ± 2.91
Relative mean change vs cluster A (%)	Reference	+23.5	+9.4	+91.8	+30.6	+57.6	+61.2
Odds of ≥1 event in cluster A patients vs other clusters, OR (*P* value)	Reference	1.507 (<.001)					
**Asthma-related hospitalizations**							
Patients with ≥1 event, n (%)	236 (11.7)	105 (13.7)	172 (13.1)	136 (17.2)	158 (14.5)	196 (17.1)	340 (14.0)
Total number of events per patient during follow-up, mean ± SD	0.22 ± 0.94	0.27 ± 0.95	0.25 ± 0.92	0.42 ± 1.31	0.32 ± 1.33	0.34 ± 1.00	0.30 ± 1.16
Relative mean change vs cluster A (%)	Reference	+22.7	+13.6	+90.9	+45.5	+54.5	+36.4
Odds of ≥1 event in cluster A patients vs other clusters, OR (*P* value)	Reference	1.306 (<.001)					
**Length of asthma-related hospital stays**							
Patients with ≥1 event, n (%)	102 (5.0)	48 (6.2)	80 (6.1)	64 (8.1)	76 (7.0)	89 (7.8)	142 (5.9)
Total number of days per patient during follow-up, mean ± SD	0.26 ± 1.54	0.41 ± 2.44	0.31 ± 1.82	0.90 ± 8.31	0.39 ± 2.09	0.51 ± 2.78	0.37 ± 2.33
Relative mean change vs cluster A (%)	Reference	+57.7	+19.2	+246.2	+50.0	+96.2	+42.3
Average difference between cluster A patients and other clusters (*P* value)	Reference	0.188 (.008)					
**Asthma-related ED visits**							
Patients with ≥1 event, n (%)	167 (8.3)	75 (9.8)	109 (8.3)	94 (11.9)	99 (9.1)	142 (12.4)	246 (10.2)
Total number of events per patient during follow-up, mean ± SD	0.16 ± 0.85	0.20 ± 0.88	0.17 ± 0.79	0.29 ± 1.09	0.23 ± 1.12	0.24 ± 0.80	0.23 ± 1.07
Relative mean change vs cluster A (%)	Reference	+25.0	+6.3	+81.3	+43.8	+50.0	+43.8
Odds of ≥1 event in cluster A patients vs other clusters, OR (*P* value)	Reference	1.258 (.010)					

*OR*, Odds ratio.

#### Health care costs

Event-specific health care costs reflected the patterns observed in exacerbations and HCRU across adherence groups (Table V). Adherent patients had the lowest all-cause costs (mean, $4,472), which increased by 12.0% in the partially adherent group, 19.8% in the minimally adherent group, and 36.3% in the treatment discontinuation group. Total inpatient costs showed a similar trend, as did total outpatient costs and asthma-related costs (Table V). These data were supported by adherence cluster analyses, confirming that higher adherence to biologics was generally associated with lower health care costs (Table VI). Notably, cluster A incurred consistently lower costs across all events versus the other clusters, with the exception of cluster B; over 12-month follow-up, total all-cause and asthma-related costs per patient (and individual cost types) were generally lower for cluster A versus all other clusters.

**Table V:** Summary of health care costs by asthma biologic treatment adherence categories during the 12-month follow-up period (N = 10,088)

Outcome	Adherent (n = 2,000)	Partially adherent (n = 2,045)	Minimally adherent (n = 528)	Treatment discontinuation (n = 5,515)
**All-cause total event-specific costs**				
Patients with ≥1 cost, n (%)	1,398 (69.9)	1,463 (71.5)	386 (73.1)	4,152 (75.3)
Cost ($) per patient, mean ± SD	4,472 ± 12,906	5,008 ± 14,271	5,358 ± 19,929	6,096 ± 15,589
Relative mean change∗ (%)	Reference	+12.0	+19.8	+36.3
Average difference between adherent patients and other categories (*P* value)	Reference	1,301.17 (<.001)		
**All-cause inpatient costs**				
Patients with ≥1 cost, n (%)	343 (17.2)	406 (19.9)	115 (21.8)	1,247 (22.6)
Cost ($) per patient, mean ± SD	1,444 ± 7,449	1,732 ± 9,421	2,374 ± 17,811	2,009 ± 9,847
Relative mean change∗ (%)	Reference	+19.9	+64.4	+39.1
Average difference between adherent patients and other categories (*P* value)	Reference	518.37 (<.001)		
**All-cause outpatient costs**				
Patients with ≥1 cost, n (%)	1,384 (69.2)	1,439 (70.4)	383 (72.5)	4,106 (74.5)
Cost ($) per patient, mean ± SD	3,526 ± 8,965	3,966 ± 11,421	3,399 ± 6,700	4,343 ± 10,715
Relative mean change∗ (%)	Reference	+12.5	−3.6	+23.2
Average difference in cost between adherent patients and other categories (*P* value)	Reference	659.93 (<.001)		
**All-cause unscheduled/ED costs**				
Patients with ≥1 cost, n (%)	576 (28.8)	673 (32.9)	192 (36.4)	2,114 (38.3)
Cost ($) per patient, mean ± SD	357 ± 1,414	410 ± 1,243	388 ± 1,081	537 ± 1,441
Relative mean change∗ (%)	Reference	+15.0	+8.7	+50.4
Average difference between adherent patients and other categories (*P* value)	Reference	138.21 (<.001)		
**All-cause pharmacy costs**				
Patients with ≥1 cost, n (%)	7 (0.4)	8 (0.4)	0	14 (0.3)
Cost ($) per patient, mean ± SD	4 ± 95	5 ± 145	0	7 ± 394
Relative mean change∗ (%)	Reference	+29.0	−100.0	+92.0
Average difference between adherent patients and other categories (*P* value)	Reference	2.47 (.831)		
**All-cause patient co-pay amount costs**				
Patients with ≥1 cost, n (%)	1,749 (87.5)	1,868 (91.3)	463 (87.7)	4,974 (90.2)
Cost ($) per patient, mean ± SD	122 ± 564	139 ± 506	159 ± 621	131 ± 590
Relative mean change∗ (%)	Reference	+13.2	+30.3	+7.2
Average difference between adherent patients and other categories (*P* value)	Reference	7.92 (.721)		
**Asthma-related total cost**				
Patients with ≥1 cost, n (%)	714 (35.7)	779 (38.1)	199 (37.7)	2,160 (39.2)
Cost ($) per patient, mean ± SD	958 ± 4,184	1,254 ± 6,745	1,667 ± 11,265	1,520 ± 7,442
Relative mean change∗ (%)	Reference	+31.0	+74.0	+58.7
Average difference between adherent patients and other categories (*P* value)	Reference	504.44 (.008)		
**Asthma-related inpatient costs**				
Patients with ≥1 cost, n (%)	191 (9.6)	226 (11.1)	68 (12.9)	648 (11.7)
Cost ($) per patient, mean ± SD	754 ± 4,306	951 ± 6,881	1,316 ± 10,927	888 ± 6,674
Relative mean change∗ (%)	Reference	+26.1	+74.5	+17.8
Average difference between adherent patients and other categories (*P* value)	Reference	178.28 (.007)		
**Asthma-related outpatient costs**				
Patients with ≥1 cost, n (%)	676 (33.8)	742 (36.3)	184 (34.8)	2,034 (36.9)
Cost ($) per patient, mean ± SD	609 ± 2,576	765 ± 3,498	664 ± 2,498	802 ± 3,883
Relative mean change∗ (%)	Reference	+25.5	+8.9	+31.6
Average difference between adherent patients and other categories (*P* value)	Reference	174.00 (.023)		
**Asthma-related unscheduled/ED costs**				
Patients with ≥1 cost, n (%)	175 (8.8)	224 (11.0)	61 (11.6)	679 (12.3)
Cost ($) per patient, mean ± SD	98 ± 855	113 ± 569	94 ± 454	132 ± 630
Relative mean change∗ (%)	Reference	+15.5	−4.6	+34.6
Average difference between adherent patients and other categories (*P* value)	Reference	26.73 (<.001)		

∗ Versus the adherent category.

**Table VI:** Summary of health care costs across 7 GBTM clusters among patients who remained on the same biologic during the 12-month follow-up period (N = 9,553)

Clinical outcomes	Cluster A (highest adherent) (n = 2,024)	Cluster B (late-stage stopper) (n = 769)	Cluster C (intermittent adherence) (n = 1,315)	Cluster D (half-way stopper) (n = 791)	Cluster E (fluctuation stopper) (n = 1,090)	Cluster F (delayed stopper) (n = 1,144)	Cluster G (lowest adherent) (n = 2,420)
**All-cause total event-specific costs**							
Patients with ≥1 event/cost, n (%)	1,434 (70.8)	562 (73.1)	925 (70.3)	609 (77.0)	825 (75.7)	879 (76.8)	1,758 (72.6)
Total cost ($) per patient during follow-up, mean ± SD	5,582.64 ± 13,958.63	5,527.10 ± 14,545.93	7,005.59 ± 33,756.58	7,056.51 ± 16,403.08	6,812.92 ± 16,923.14	7,125.59 ± 20,467.18	7,544.50 ± 22,165.67
Relative mean change vs cluster A (%)	Reference	−1.0	+25.5	+26.4	+22.0	+27.6	+35.1
Average difference between cluster A patients and other clusters (*P* value)	Reference	1,440.66 (<.001)					
**All-cause inpatient costs**							
Patients with ≥1 event/cost, n (%)	324 (16.0)	139 (18.1)	265 (20.2)	196 (24.8)	259 (23.8)	276 (24.1)	522 (21.6)
Total cost ($) per patient during follow-up, mean ± SD	1,337.36 ± 6,484.69	2,173.73 ± 11,701.49	2,518.10 ± 19,299.82	2,391.28 ± 10,938.33	2,454.71 ± 10,944.12	2,117.09 ± 9,262.64	2,202.49 ± 14,564.02
Relative mean change vs cluster A (%)	Reference	+62.5	+88.3	+78.8	+83.5	+58.3	+64.7
Average difference between cluster A patients and other clusters (*P* value)	Reference	960.69 (<.001)					
**All-cause outpatient costs**							
Patients with ≥1 event/cost, n (%)	1,420 (70.2)	555 (72.2)	913 (69.4)	596 (75.3)	814 (74.7)	872 (76.2)	1,742 (71.2)
Total cost ($) per patient during follow-up, mean ± SD	4,676.57 ± 11,770.91	4,102.67 ± 8,616.60	5,381.80 ± 22,662.48	5,112.89 ± 11,147.82	4,950.05 ± 11,583.38	5,213.76 ± 17,370.34	5,459.08 ± 14,585.16
Relative mean change vs cluster A (%)	Reference	−12.3	+15.1	+9.3	+5.8	+11.5	+16.7
Average difference between cluster A patients and other clusters (*P* value)	Reference	483.13 (.014)					
**All-cause unscheduled/ED costs**							
Patients with ≥1 event/cost, n (%)	587 (29.0)	250 (32.5)	437 (33.2)	311 (39.3)	382 (35.0)	449 (39.2)	936 (38.7)
Total cost ($) per patient during follow-up, mean ± SD	513.75 ± 2,394.51	533.75 ± 1,831.80	580.99 ± 2,096.58	892.15 ± 2,415.60	596.22 ± 2,089.23	764.42 ± 2,428.20	726.48 ± 2,303.99
Relative mean change vs cluster A (%)	Reference	+3.9	+13.1	+73.7	+16.1	+48.8	+41.4
Average difference between cluster A patients and other clusters (*P* value)	Reference	171.94 (<.001)					
**All-cause pharmacy costs**							
Patients with ≥1 cost, n (%)	7 (0.4)	1 (0.1)	6 (0.5)	3 (0.4)	2 (0.2)	5 (0.4)	3 (0.1)
Cost ($) per patient, mean ± SD	5.62 ± 151.34	0.02 ± 0.50	5.01 ± 105.94	1.55 ± 29.34	0.18 ± 4.31	34.42 ± 863.60	0.30 ± 9.50
Relative mean change∗ (%)	Reference	−99.6	−10.9	−72.4	−96.8	+512.5	−94.7
Average difference between cluster A patients and other clusters (*P* value)	Reference	0.77 (.545)					
**All-cause patient co-pay amount costs**							
Patients with ≥1 cost, n (%)	259 (12.8)	125 (16.3)	246 (18.7)	137 (17.3)	164 (15.1)	142 (12.4)	247 (10.2)
Cost ($) per patient, mean ± SD	140.43 ± 667.55	151.20 ± 604.50	166.85 ± 771.57	156.55 ± 590.89	150.80 ± 566.54	146.79 ± 618.80	141.29 ± 696.50
Relative mean change∗ (%)	Reference	+7.7	+18.8	+11.5	+7.4	+4.5	+0.6
Average difference between cluster A patients and other clusters (*P* value)	Reference	10.15 (.115)					
**Asthma-related total cost**							
Patients with ≥1 cost, n (%)	720 (35.6)	287 (37.3)	485 (36.9)	320 (40.5)	405 (37.2)	467 (40.8)	923 (38.1)
Total cost ($) per patient during follow-up, mean ± SD	1,377.26 ± 5,887.23	1,671.31 ± 9,268.13	1,645.39 ± 6,286.68	1,699.47 ± 7,037.76	1,844.98 ± 7,677.31	2,006.33 ± 8,152.28	1,942.33 ± 11,354.37
Relative mean change vs cluster A (%)	Reference	+21.4	+19.5	+23.4	+34.0	+45.7	+41.0
Average difference between cluster A patients and other clusters (*P* value)	Reference	455.64 (.011)					
**Asthma-related inpatient cost**							
Patients with ≥1 cost, n (%)	170 (8.4)	81 (10.5)	147 (11.2)	104 (13.1)	146 (13.4)	156 (13.6)	252 (10.4)
Total cost ($) per patient during follow-up, mean ± SD	764.54 ± 5,016.54	1,429.66 ± 10,508.71	1,136.41 ± 6,389.26	978.36 ± 6,964.72	1,363.31 ± 7,868.35	1,028.43 ± 6,735.18	950.70 ± 9,828.30
Relative mean change vs cluster A (%)	Reference	+87.0	+48.6	+28.0	+78.3	+34.5	+24.3
Average difference between cluster A patients and other clusters (*P* value)	Reference	341.97 (<.001)					
**Asthma-related outpatient cost**							
Patients with ≥1 cost, n (%)	690 (34.1)	276 (35.9)	458 (34.8)	304 (38.4)	374 (34.3)	436 (38.1)	866 (35.8)
Total cost ($) per patient during follow-up, mean ± SD	965.38 ± 4,372.74	825.57 ± 2,840.49	1,073.26 ± 4,235.95	1,082.93 ± 3,833.22	975.12 ± 3,274.88	1,085.71 ± 4,297.09	1,052.84 ± 5,312.01
Relative mean change vs cluster A (%)	Reference	−14.5	+11.2	+12.2	+10.1	+12.5	+9.1
Average difference between cluster A patients and other clusters (*P* value)	Reference	64.72 (.081)					
**Asthma-related unscheduled/ED cost**							
Patients with ≥1 cost, n (%)	179 (8.8)	78 (10.1)	139 (10.6)	102 (12.9)	116 (10.6)	155 (13.5)	282 (11.7)
Total cost ($) per patient during follow-up, mean ± SD	163.50 ± 1,350.50	153.80 ± 845.71	200.99 ± 1,525.72	301.97 ± 1,343.71	173.52 ± 1,024.85	237.05 ± 1,360.98	216.13 ± 1,319.88
Relative mean change vs cluster A (%)	Reference	−5.9	+22.9	+84.7	+6.1	+45.0	+32.2
Average difference between cluster A patients and other clusters (*P* value)	Reference	49.65 (<.001)					

∗ Versus the adherent category.

### Impact of COVID-19

Before March 1, 2020 (before the start of the COVID-19 pandemic), 5,288 patients had completed their 12-month follow-up period. After this date, 2,569 patients were still being followed. Overall, the average MPR decreased slightly from 43.8% pre-COVID to 41.2% post-COVID (for full details, see Table E4 in this article’s Online Repository at www.jaci-global.org). A summary of asthma-related ED visits, hospitalizations, and corticosteroid use before and after the COVID-19 pandemic is provided in Table E5 (in the Online Repository available at www.jaci-global.org).

## Discussion

This 12-month retrospective, observational, real-world evidence study demonstrated that adherence to biologic therapies in patients with severe asthma was associated with better clinical outcomes, decreased HCRU, and lower overall health care costs compared with those with lower adherence to biologics. Patients who were adherent to their biologics experienced fewer hospitalization-, ED-, and corticosteroid-related exacerbations. The positive impact of adherence was consistent across most clinical, HCRU, and health care cost–related outcomes, reinforcing its critical role in optimizing treatment effectiveness and efficiency. These findings highlight the importance of considering adherence to labeled dosing when it comes to treatment strategies, including topics such as biologic selection. The use of GBTM enabled the identification of distinct adherence clusters, which may help clinicians to identify patients at risk of poor clinical outcomes in real-world practice and inform the implementation of targeted interventions. In addition, the economic findings of this study emphasize the cost-saving potential of improved adherence to biologic therapies, supporting the case for adherence-enhancing strategies such as patient education and digital support tools (which have been identified as important to maintaining high biologic adherence for patients with asthma).^23^ The development of biologics with sustained efficacy may also support adherent behaviors, given that more frequent dosing has been linked to lower adherence.^20^

The results of this study are consistent with those of previous studies demonstrating the substantial impact of adherence on the effectiveness and cost-effectiveness of biologics and/or standard-of-care therapies.^1^^,^^22^^,^^24^, ^25^, ^26^ Notably, a systematic review of 23 high-quality clinical studies found that adherence to asthma controller medication (ICSs, LABAs, or fixed-combination ICS/LABA therapy) was associated with fewer severe asthma exacerbations,^24^ aligning with the outcomes observed in this study. In addition, this study demonstrated no clear pattern in SABA use by adherence level, suggesting that SABA use may be less sensitive to adherence levels than other exacerbation-related outcomes. However, adherence to biologics in the present study was poor, with more than half the patients discontinuing treatment during the 12-month follow-up. This contrasts with earlier findings showing higher adherence to biologics compared with ICSs, both before and after biologic initiation.^21^ The lower adherence observed here may be partly explained by the study’s longer follow-up period and more stringent definition of adherence. As previously noted, there is considerable variation in how adherence is assessed; strategies include measuring the proportion of missed doses, days covered or patients covered, as well as ratio of administered doses to expected doses, MPR, or health care providers’ perceptions of adherence.^22^ For example, proportion of days covered (PDC) may cover only shorter time frames (eg, a PDC of 0.76 in the first 6 months of biologic treatment was reported in a similar retrospective claims data study and a PDC of 86% in the first year of biologic use).^21^^,^^23^ The dose- and gap-based categorization approach used in the present study was developed to better reflect the specific labeled dosing intervals and real-world treatment dynamics of asthma biologics, and it enables researchers to distinguish between different adherence behaviors rather than relying solely on a summary percentage. There is existing evidence from nonasthma indications that biologics, such as dupilumab and benralizumab, are less effective when administered on an extended-dosing schedule, resulting in clinical worsening.^27^^,^^28^ These earlier findings support the dose- and gap-based approach to adherence categorization used in the present study. The longer time frame and stricter adherence definition in this study also offer unique insights into long-term adherence behavior and its implications for both clinical, HCRU, and economic outcomes. Although this approach may appear to yield lower adherence rates compared with traditional PDC methods, it also provides a more nuanced and clinically relevant understanding of patient adherence in the context of biologic therapies. It should also be noted that adherence tends to be higher with intravenously or subcutaneously administered biologics compared with oral therapies, on the basis of previous studies.^21^^,^^29^ These findings underscore the need and opportunity for improved adherence to asthma biologics, further supporting investment in strategies to enhance long-term treatment adherence.

The COVID-19 pandemic resulted in substantial reductions in primary exacerbation outcomes, such as asthma-related hospital admissions, use of oral and intravenous corticosteroids, and ED visits for asthma,^30^ which may reflect factors such as reduced viral infections caused by a decrease in social interactions and changes in patient behavior. To address these uncertainties, this study examined patterns of exacerbations before and after March 2020, a period when the pandemic markedly changed health care interactions in the United States. Average MPR decreased only slightly for the overall population post-COVID and remained relatively stable or slightly improved post-COVID in the high-adherence clusters, and the proportion of patients with exacerbation-related outcomes decreased overall and across most of the clusters after the COVID-19 pandemic (compared with before March 2020).

Strengths of this study include the evaluation of all 6 biologics approved for the treatment of asthma, its large sample size, and the long time period over which data were captured (2007-2023). In addition, adherence was comprehensively investigated using MPR analysis and GBTM analysis, with adherence categories defined separately for each individual biologic, and the observed trends are consistent with results obtained in another real-world study using the Komodo Research Database,^31^ supporting the generalizability of our findings. Like all real-world studies, this observational study also has some limitations. The Optum Market Clarity data set, although extensive, is not fully representative of the entire US population, may not fully encompass underinsured populations, and does not capture adherence patterns beyond the first year of treatment. EHR data are collected primarily for patient management and so do not directly reflect medication intake. EHR data may also be incomplete, and reasons for discontinuing biologic therapy may not be available or may be misclassified. Linking EHR and claims data can also introduce selection bias. In addition, although claims data offer detailed insights into HCRU and prescriptions issued, data on biologics outside the capture period, self-funded prescriptions, or prescriptions paid outside the primary insurer would not be included, potentially leading to underestimation of health care costs and outcomes. Pharmacy claims also include assumptions that patients take their medications as prescribed. Similarly, variations in the pharmacokinetic characteristics of biologics could also affect the assessment of adherence given the 7-day buffer applicable to all treatments. Applying a uniform 7-day buffer across all biologics may not fully account for differences in pharmacokinetics, particularly half-life and dosing schedules. Future sensitivity analyses may tailor adherence definitions to the pharmacokinetic profiles and dosing intervals of each biologic. Another key consideration is the predominance of omalizumab in the study population, which likely reflects its status as the first biologic approved for severe asthma.^32^ The study design’s requirement for eligible patients to be biologic-naive before the index date, as well as the exclusion of patients who switched treatments (GBTM analysis only), further limited the representation of more recently approved biologics such as reslizumab and tezepelumab and did not reflect subsequent treatment changes. Irrespective of these limitations, the consistency of findings using treatment adherence categorization and GBTM analyses, as well as the sensitivity and variability of the GBTM analysis shown here, supports the robustness of the findings. Alternative methods, such as latent class analysis, could complement these results and further validate the findings.

In conclusion, these data show that adherence to current biologics was suboptimal, and poor adherence was associated with worse clinical outcomes, increased HCRU, and health care costs. These data offer valuable insights for clinicians, payers, and policymakers alike, and the identification of distinct adherence patterns provides a framework for targeted interventions to improve both adherence and patient outcomes. The potential cost savings associated with adherence also support investment in adherence-enhancing strategies.

## Disclosure statement

Study 214570 is a GSK-sponsored study. The sponsor was involved in study design and implementation, as well as data collection, analysis, interpretation, writing the study report and reviewing this manuscript. All authors had full access to the data upon request and had final responsibility for the decision to submit for publication.

Disclosure of potential conflict of interest: N. L. Lugogo received consulting fees from Amgen, AstraZeneca, Avillion, Genentech, GSK, Niox, Novartis, Regeneron, Sanofi, and Teva; honoraria for nonspeakers bureau presentations from GSK, Teva, and AstraZeneca; received travel support from AstraZeneca, Sanofi, Teva, Regeneron, and GSK; received research support (through her institution) from Amgen, AstraZeneca, Avillion, Bellus, Evidera, Gossamer Bio, Genentech, GSK, Janssen, Niox, Regeneron, Sanofi, Novartis, and Teva; and is an honorary faculty member of Observational and Pragmatic Research Institute but does not receive compensation for this role. B. Modena received research funding from GSK as part of a multicenter investigator-supported study. R. Al-Lehebi has given lectures at meetings supported by AstraZeneca, Boehringer Ingelheim, GSK, and Sanofi; and received advisory board fees from GSK, Sanofi, and AstraZeneca. J. Wang and G. Smith are employees of Cytel, Inc, which received funding from GSK for this study. R. Geason was an employee of Cytel, Inc at the time of the study, which received funding from GSK for this study. J. Kwiatek, P. Howarth, A. Vichiendilokkul, U. Karsanji, J. Hwee, and A. Deb are employees of GSK and hold financial equities in GSK. R. Alfonso-Cristancho was an employee of GSK at the time of the study and holds financial equities in GSK.
