# Supplementary material for: Consistent adherence to biologic therapies is critical for improving outcomes in asthma
Source: J Allergy Clin Immunol Glob. 2026 Aug 7;5(6):100771. doi: 10.1016/j.jacig.2026.100771 (PMC13581996; doi:10.1016/j.jacig.2026.100771)
Supplement: Supplementary file 1 [file mmc1.docx]

**ONLINE REPOSITORY**

**Plain language summary**

Some people with asthma continue to have asthma attacks even when they take their usual asthma medicines. In these cases, doctors may prescribe an additional treatment called a biological therapy (a ‘biologic’). In everyday clinical practice (outside of clinical trials), there is limited information about what happens to asthma symptoms when people stop taking their biologic. We do know that biologics work best when they are taken exactly as prescribed – this is called adherence. In this GSK-funded study, researchers used electronic health records to look at information from adults with asthma in the United States who received any biologic therapy for asthma between 2007 and 2023. The team measured how regularly people collected their biologic therapy over 1 year. This was called the medication possession ratio (MPR), which gives an estimate on how closely someone followed their prescribed treatment. The team also used a type of analysis called group-based trajectory modeling (GBTM) to understand patterns, such as when people missed doses or stopped their treatment altogether. After identifying these patterns, the team investigated whether asthma attacks, use of healthcare resources, and healthcare costs differed between people who took their biologic as prescribed and those who were less adherent.

Overall, 10,088 patients were included. More than half stopped their biologic therapy earlier than expected; the rest showed different levels of adherence. The MPR analysis looked at 8151 people who took at least one additional dose after starting their biologic. In this group, between 41% and 64% of people were considered adherent. The GBTM analysis found seven different patterns of how people took their biologic over time, including high adherence, low adherence, and patterns where adherence changed during the year. Overall, people who took the biologic as prescribed had better outcomes. They had fewer asthma attacks, used fewer healthcare services, and had lower healthcare costs compared with those who were less adherent to their biologic therapy. These results highlight the importance of biologic adherence for severe asthma management.

**Methods**

Detailed exclusion criteria

Patients must have received ≥1 prescription for both ICS and long-acting β_2_-agonist medication (LABA; either alone or in combination with long-acting muscarinic antagonists [LAMA]) within the 90 days prior to and including the latest asthma diagnosis date for inclusion in the study.

Patients were excluded if they had any evidence of biologic prescriptions from a dermatologist at any time, based on physician specialty information from either EHR or claims databases (this criterion was used to minimize the possibility that prior biologic use for non-asthma indications could influence adherence). Patients with a documented diagnosis of lung malignancies, active respiratory tuberculosis, or cystic fibrosis at any time prior to index, as identified through ICD-9-CM and ICD-10-CM codes, were also excluded. Patients diagnosed with chronic spontaneous urticaria or atopic dermatitis within ±7 days of index were excluded to avoid confounding due to shared immunologic pathways, symptom overlap, potential medication interference, or other disease outcomes that could influence biologic adherence. Further exclusions included patients who received prescriptions for more than one biologic on index, those with missing or unavailable data on patient sex, and those diagnosed with hypereosinophilic syndrome or eosinophilic granulomatosis with polyangiitis at any point before the end of the follow-up period.

Clinical outcomes

Other outcomes included SABA use, frequency and proportion of patients with HCRU (including all-cause and asthma-related inpatient admissions, ED visits, and outpatient encounters), and event-specific healthcare costs (reported in US dollars) for all-cause and asthma-related events (inpatient, outpatient, ED, pharmacy, and patient copay) across treatment adherence categories and clusters during the follow-up period. Biologic administration-related outpatient events and associated costs (not including the cost of the biologic) were excluded from the extraction of exacerbation, HCRU, and event-specific cost outcomes.

COVID analysis

The impact of the COVID-19 pandemic on asthma exacerbations was assessed as an exploratory objective by analyzing differences in exacerbation number and the percentage of patients experiencing exacerbations before/after March 2020 across different treatment adherence groups.

Statistical analysis

Descriptive analyses and heatmaps first assessed missed dose frequency for each biologic based on binary adherence. Longitudinal adherence clusters, which were not stratified by specific biologics, were subsequently identified through GBTM, which was applied to patients who remained on the same biologic during follow-up. Binary adherence over time was modeled using logistic regression with polynomial terms to capture trajectory variations, and model fit was evaluated using Akaike and Bayesian information criterion. Primary clinical (exacerbations), HCRU, and cost outcomes were evaluated across adherence clusters using descriptive and statistical analyses. Exacerbation frequency and patient proportions were examined over 12 months for three single exacerbation events across the adherence categories and GBTM clusters.

Count data (exacerbations, HCRU) were analyzed using Poisson regression initially, and negative binomial regression was used if overdispersion was detected. Logistic regression was used to assess if there were significant differences among clusters in proportion of patients with at least one exacerbation or HCRU event. Odds ratios were reported to quantify the association between adherence and the likelihood of experiencing each outcome. Average differences were reported to quantify differences of costs or lengths of hospital stays between adherent and patients in other categories and between Cluster A and patients in other clusters. For continuous outcomes like costs, one-way analysis of variance or Kruskal–Wallis tests (if non-normal) assessed mean differences. Post hoc pairwise comparisons used Bonferroni adjustments for continuous variables and Tukey adjustments for count and categorical outcomes*.*

**e-Figure 1. Average adherence for patients receiving one of six approved asthma biologics during 12 months of follow-up (MPR analysis)**

MPR, medication possession ratio.

**e-Figure 2. Heatmaps illustrating treatment adherence patterns for each approved asthma biologic during the 12-month follow-up period**

The heatmaps indicate frequent interruptions and restarts in treatment regimens. Gaps in

adherence were common, particularly when treatments occurred outside the scheduled

timeline, even with a ±7-day buffer around the dynamic expected treatment date. Doses

administered beyond this range were not captured, potentially underestimating adherence

levels.

**e-Table 1. Treatment adherence categorization for each approved asthma biologic over the 12-month follow-up period^a^**

|  | Adherent | Partially  adherent | Minimally adherent | Treatment discontinuation |
| --- | --- | --- | --- | --- |
| Benralizumab  Q4W for the first 3 doses, then Q8W | | | | |
| Treatment gap to be evaluated between first and second dose | All gaps <56 days AND >4 doses | ≥1 gap ≥56 days with >4 doses OR ≤4 doses with all gaps <56 days | ≥1 gap ≥56 days AND  ≤4 doses | ≥112-day gap at the end while remaining active in dataset |
| Treatment gap to be evaluated between second and third dose | All gaps <84 days AND >4 doses | ≥1 gap ≥84 days with >4 doses OR ≤4 doses with all gaps <84 days | ≥1 gap ≥84 days AND  ≤4 doses | ≥140-day gap at the end while remaining active in dataset |
| Treatment gap to be evaluated any time after third dose | All gaps <112 days AND  >4 doses | ≥1 gap ≥112 days with >4 doses OR ≤4 doses with all gaps <112 days | ≥1 gap ≥112 days AND  ≤4 doses | ≥168-day gap at the end while remaining active in dataset |
| Dupilumab | | | | |
| Q2W | <28-day gap and  >13 doses | ≥28-day gap and >13 doses  OR  <28-day gap and ≤13 doses | ≥28-day gap and ≤13 doses | ≥42-day gap at the end of 12 months while remaining active in data^b^ |
| Mepolizumab | | | | |
| Q4W | <56-day gap and >7 doses | ≥56-day gap and  >7 doses  OR  <56-day gap and  ≤7 doses | ≥56-day gap and ≤7 doses | ≥84-day gap at the end of 12 months while remaining active in dataset |
| Omalizumab | | | | |
| Q2W | <28-day gap and >13 doses | ≥28-day gap with >13 doses  OR  <28-day gap and ≤13 doses | ≥28-day gap and ≤13 doses | ≥42-day gap at the end of 12 months while remaining active in dataset |
| Q4W | <56-day gap and  >7 doses | ≥56-day gap and  >7 doses  OR  with <56-day gap and ≤7 doses | ≥56-day gap over and  ≤7 doses | ≥84-day gap at the end of 12 months while remaining active in dataset |
| Reslizumab | | | | |
| Q4W | <56-day gap and >7 doses | ≥56-day gap and  >7 doses OR <56-day gap and  ≤7 doses | ≥56-day gap and ≤7 doses | ≥84-day gap at the end of 12 months while remaining active in dataset |
| Tezepelumab | | | | |
| Q4W | <56-day gap and >7 doses | ≥56-day gap and  >7 doses OR ≤7 doses and  <56-day gap | ≥56-day gap and ≤7 doses | ≥84-day gap at the end of 12 months while remaining active in dataset |

^a^If a patient met the criteria for both treatment discontinuation and potential adherent, partially adherent, or minimally adherent simultaneously, the patient was categorized as treatment discontinuation first. ^b^The patient meets the eligibility criteria by being present in the dataset for the full 12-month follow-up. However, there is a ≥42-day gap without any biologic administration leading up to the end of that follow-up window. This gap is used to flag treatment discontinuation, as it suggests the patient stopped receiving biologic therapy, even though we can still track them in the data.
QxW, every x weeks.

**e-Table 2. Baseline patient characteristics stratified by asthma biologic (N=10,088)**

|  | Benralizumab  (n=1301) | Dupilumab  (n=2096) | Mepolizumab  (n=1545) | Omalizumab  (n=5048) | Reslizumab  (n=84) | Tezepelumab  (n=14) | SMD |
| --- | --- | --- | --- | --- | --- | --- | --- |
| Age, in years, median (IQR) | 59.6  (49.28, 67.23) | 57.7  (45.71, 65.68) | 58.5  (49.16, 66.85) | 54.6  (43.70, 63.06) | 56.5  (44.87, 62.48) | 47.7  (40.97, 57.82) | 0.267 |
| Sex, % | | | | | | | |
| Male | 33.8 | 36.4 | 35.8 | 34.8 | 33.3 | 21.4 | 0.123 |
| Female | 66.2 | 63.6 | 64.2 | 65.2 | 66.7 | 78.6 |  |
| Race, % | | | | | | | |
| Black/African American | 13.8 | 12.5 | 13.3 | 15.2 | 7.1 | 14.3 | 0.179 |
| Asian | 1.5 | 2.9 | 1.4 | 1.7 | 1.2 | 0.0 |  |
| White | 72.7 | 73.9 | 75.5 | 70.9 | 81.0 | 71.4 |  |
| Other/unknown | 12.1 | 10.8 | 9.8 | 12.2 | 10.7 | 14.3 |  |
| Ethnicity, % | | | | | | | |
| Hispanic | 6.1 | 4.9 | 6.6 | 5.1 | 1.2 | 7.1 | 0.207 |
| Not Hispanic | 77.9 | 78.8 | 80.3 | 78.7 | 77.4 | 85.7 |  |
| Other/Unknown | 15.9 | 16.3 | 13.1 | 16.2 | 21.4 | 7.1 |  |
| Region, % | | | | | | | |
| Midwest | 28.9 | 36.1 | 40.1 | 38.9 | 41.7 | 28.6 | 0.224 |
| Northeast | 29.0 | 24.6 | 28.7 | 23.6 | 19.0 | 28.6 |  |
| South | 26.1 | 21.6 | 18.9 | 21.6 | 22.6 | 21.4 |  |
| West | 12.3 | 13.1 | 8.1 | 11.4 | 9.5 | 14.3 |  |
| Other/unknown | 3.8 | 4.6 | 4.2 | 4.5 | 7.1 | 7.1 |  |
| Median (range) index year | 2019  (2019, 2020) | 2020  (2019, 2021) | 2018  (2017, 2020) | 2016  (2014, 2018) | 2017.5  (2017, 2019) | 2022  (2022, 2022) | 1.833 |
| Smoking status, % | | | | | | | |
| Current | 6.0 | 5.3 | 6.4 | 5.7 | 4.8 | 14.3 | 0.485 |
| Former | 23.8 | 20.0 | 23.7 | 14.5 | 16.7 | 14.3 |  |
| Never | 37.0 | 37.0 | 33.7 | 29.3 | 28.6 | 64.3 |  |
| Other/unknown | 33.2 | 33.2 | 36.2 | 50.5 | 50.0 | 7.1 |  |
| Bodyweight based on BMI, % | | | | | | | |
| Underweight | 0.7 | 0.8 | 1.0 | 0.3 | 2.4 | 0.0 | 0.367 |
| Normal | 10.8 | 11.5 | 9.3 | 8.3 | 3.6 | 14.3 |  |
| Overweight | 18.4 | 18.7 | 19.1 | 15.7 | 10.7 | 14.3 |  |
| Obese | 44.1 | 41.3 | 41.7 | 33.6 | 42.9 | 57.1 |  |
| Unknown | 26.0 | 27.8 | 28.8 | 42.1 | 40.5 | 14.3 |  |
| Insurance type, % | | | | | | | |
| Commercial | 45.5 | 48.0 | 42.1 | 39.2 | 42.9 | 42.9 | 0.432 |
| Medicaid | 8.9 | 6.6 | 8.3 | 8.1 | 3.6 | 14.3 |  |
| Medicare | 19.1 | 17.4 | 20.1 | 11.1 | 15.5 | 28.6 |  |
| Other payer | 1.6 | 1.1 | 1.4 | 1.0 | 3.6 | 7.1 |  |
| Uninsured | 1.0 | 0.7 | 1.2 | 1.2 | 3.6 | 0.0 |  |
| Unknown | 23.8 | 26.1 | 27.0 | 39.4 | 31.0 | 7.1 |  |
| Weighted CCI, median (IQR) | 5  (0, 13) | 3  (0, 9) | 4  (0, 14) | 1  (0, 8) | 2  (0, 8.25) | 7  (6, 9.75) | 0.138 |
| BEC ^a^, cells/μL, median (IQR) | 300.00  (110.00, 510.00) | 200.00  (100.00, 400.00) | 300.00  (160.00, 590.00) | 200.00  (100.00, 370.00) | 400.00  (137.50, 640.00) | 115.00  (100.00, 212.50) | 0.186 |
| IgE level^b^, IU/ml, median (IQR) | 108.00  (31.75, 408.95) | 101.00  (24.99, 343.00) | 86.00  (27.00, 276.00 | 243.00  (100.00, 549.25) | 106.85  (28.62, 367.75) | 65.00  (30.50, 157.50) | 0.142 |
| FEV_1_^c^, liters, median (IQR) | 1.54  (1.08, 1.86) | 1.54  (1.08, 1.90) | 1.55  (1.10, 1.87) | 1.62  (1.28, 1.93) | 2.10  (1.67, 2.89) | 1.60  (1.30, 1.80) | 0.367 |

Patient numbers were not available for sex, race, ethnicity, region, smoking status, BMI, and type of insurance, but the percentages of patients are provided.

^a^Among patients with ≥1 non-missing value: benralizumab, n=777; dupilumab, n=1043; mepolizumab, n=892; omalizumab, n=2099; reslizumab, n=42; tezepelumab, n=12. ^b^Among patients with ≥1 non-missing value: benralizumab, n=395; dupilumab, n=373; mepolizumab, n=475; omalizumab, n=1634; reslizumab, n=20; tezepelumab, n=8. ^c^Among patients with ≥1 non-missing value: benralizumab, n=116; dupilumab, n=91; mepolizumab, n=139; omalizumab, n=309; reslizumab, n=13; tezepelumab, n=3.
BEC, blood eosinophil count; BMI, body mass index; CCI, Charlson Comorbidity Index; FEV_1_, forced expiratory volume in 1 second; IgE, immunoglobulin E; IQR, interquartile range; SMD, standardized mean difference.

**e-Table 3. Baseline patient characteristics across seven GBTM adherence clusters among patients who remained on the same biologic during the 12-month follow-up period (N=9553)**

|  | Cluster A  Highest adherent  (n=2024) | Cluster B  Late-stage stopper  (n=769) | Cluster C  Intermittent adherence  (n=1315) | Cluster D  Half-way  stopper  (n=791) | Cluster E  Fluctuated stopper  (n=1090) | Cluster F  Delayed  stopper  (n=1144) | Cluster G  Lowest  adherent  (n=2420) | SMD |
| --- | --- | --- | --- | --- | --- | --- | --- | --- |
| Age, in years, median (IQR) | 57.8  (48.07, 65.67) | 55.0  (44.42, 64.00) | 55.4  (44.89, 63.53) | 55.2  (44.25, 62.99) | 56.3  (45.40, 65.72) | 56.6  (44.22, 64.83) | 56.3  (44.21, 65.41) | 0.078 |
| Sex, % | | | | | | | | |
| Male | 35.4 | 32.8 | 35.1 | 39.1 | 37.2 | 34.4 | 33.9 | 0.053 |
| Female | 64.6 | 67.2 | 64.9 | 60.9 | 62.8 | 65.6 | 66.1 |  |
| Race, % | | | | | | | | |
| African American | 11.3 | 13.3 | 13.4 | 15.9 | 16.6 | 11.8 | 15.8 | 0.109 |
| Asian | 1.6 | 1.8 | 1.4 | 1.9 | 2.8 | 2.2 | 1.9 |  |
| White | 76.2 | 71.7 | 71.2 | 70.3 | 69.9 | 74.1 | 72.2 |  |
| Other/unknown | 10.9 | 13.3 | 14.1 | 11.9 | 10.7 | 11.9 | 10.1 |  |
| Ethnicity, % | | | | | | | | |
| Hispanic | 4.6 | 4.6 | 5.2 | 5.3 | 5.0 | 6.0 | 6.1 | 0.075 |
| Not Hispanic | 79.9 | 78.3 | 75.4 | 79.1 | 78.3 | 78.7 | 80.9 |  |
| Other/Unknown | 15.5 | 17.2 | 19.4 | 15.5 | 16.8 | 15.3 | 13.0 |  |
| Region, % | | | | | | | | |
| Midwest | 38.4 | 36.9 | 34.3 | 37.0 | 36.3 | 38.9 | 37.7 | 0.114 |
| Northeast | 23.9 | 24.2 | 26.5 | 22.5 | 28.5 | 24.5 | 25.4 |  |
| South | 23.1 | 25.6 | 23.4 | 23.8 | 20.0 | 20.9 | 19.0 |  |
| West | 10.5 | 9.4 | 11.3 | 11.1 | 11.7 | 10.7 | 13.2 |  |
| Other/unknown | 4.1 | 3.9 | 4.6 | 5.6 | 3.5 | 5.1 | 4.7 |  |
| Median (range) index year | 2019  (2016, 2020) | 2018  (2016, 2020) | 2017  (2015, 2019) | 2018  (2015, 2020) | 2019  (2016, 2020) | 2018  (2016, 2020) | 2019  (2016, 2020) | 0.179 |
| Smoking status, % | | | | | | | | |
| Current | 5.0 | 4.3 | 4.9 | 5.2 | 5.9 | 5.3 | 7.8 | 0.227 |
| Former | 15.4 | 14.7 | 13.6 | 17.8 | 23.4 | 17.5 | 21.9 |  |
| Never | 31.7 | 30.8 | 27.0 | 26.7 | 36.1 | 34.4 | 38.3 |  |
| Other/unknown | 47.9 | 50.2 | 54.5 | 50.3 | 34.7 | 42.8 | 32.1 |  |
| Bodyweight based on BMI, % | | | | | | | | |
| Underweight | 0.5 | 0.3 | 0.5 | 0.1 | 0.9 | 0.6 | 0.6 | 0.266 |
| Normal | 8.2 | 9.5 | 7.9 | 8.5 | 11.5 | 9.2 | 10.6 |  |
| Overweight | 17.9 | 14.7 | 13.3 | 13.5 | 19.3 | 16.3 | 20.0 |  |
| Obese | 34.0 | 32.6 | 29.3 | 32.5 | 42.8 | 38.1 | 45.6 |  |
| Unknown | 39.3 | 42.9 | 49.0 | 45.4 | 25.6 | 35.8 | 23.3 |  |
| Insurance type, % | | | | | | | | |
| Commercial | 41.7 | 38.0 | 35.9 | 37.2 | 46.6 | 44.8 | 45.7 | 0.226 |
| Medicaid | 7.3 | 8.5 | 5.9 | 7.7 | 8.2 | 7.9 | 9.0 |  |
| Medicare | 13.8 | 12.2 | 12.1 | 11.8 | 17.7 | 13.2 | 17.9 |  |
| Other payer | 1.0 | 1.2 | 0.9 | 1.0 | 1.1 | 1.0 | 1.8 |  |
| Uninsured | 0.7 | 0.5 | 0.9 | 1.3 | 0.8 | 1.0 | 1.7 |  |
| Unknown | 35.5 | 39.7 | 44.3 | 41.1 | 25.6 | 32.3 | 23.8 |  |
| Weighted CCI, median (IQR) | 27  (0, 216) | 27  (0, 162) | 27  (0, 162) | 27  (0, 216) | 108  (0, 297) | 54  (0, 216) | 135  (14, 297) | 0.154 |
| BEC^a^, cells/μL, median (IQR) | 220.00  (100.00, 460.00) | 210.00  (100.00, 500.00) | 200.00  (100.00, 400.00) | 200.00  (100.00, 400.00) | 200.00  (100.00, 421.50) | 200.00  (100.00, 400.00) | 200.00  (100.00, 400.00) | 0.064 |
| IgE level^b^, IU/ml, median (IQR) | 184.00  (52.45, 539.85) | 153.50  (59.25, 406.75) | 171.00  (61.25, 440.25) | 183.80  (59.00, 497.00) | 181.05  (61.81, 487.25) | 186.05  (73.43, 552.75) | 177.00  (62.50,  445.50) | 0.066 |
| FEV_1_^c^, liters, median (IQR) | 1.62  (1.15, 2.10) | 1.64  (1.25, 2.02) | 1.57  (1.08, 1.86) | 1.64  (1.17, 1.96) | 1.60  (1.09, 1.87) | 1.60  (1.23, 1.86) | 1.57  (1.26, 1.89) | 0.125 |

Patient numbers were not available for sex, race, ethnicity, region, smoking status, BMI, and type of insurance, but the percentages of patients are provided.

^a^Among patients with ≥1 non-missing value: Cluster A, n=840; Cluster B, n=307; Cluster C, n=503; Cluster D, n=344; Cluster E, n=607; Cluster F, n=544 Cluster G, n=1393. ^b^Among patients with ≥1 non-missing value: Cluster A, n=491; Cluster B, n=166; Cluster C, n=326; Cluster D, n=203; Cluster E, n=372; Cluster F, n=316; Cluster G, n=807. ^c^Among patients with ≥1 non-missing value: Cluster A, n=102; Cluster B, n=46; Cluster C, n=63; Cluster D, n=51; Cluster E, n=73; Cluster F, n=56; Cluster G, n=236.

BEC, blood eosinophil count; BMI, body mass index; CCI, Charlson Comorbidity Index; FEV_1_, forced expiratory volume in 1 second; GBTM, group-based trajectory modeling; IgE, immunoglobulin E; IQR, interquartile range; SMD, standardized mean difference.

**e-Table 4. Treatment adherence across seven GBTM clusters by COVID-19 period**

|  |  | **Cluster A** | **Cluster B** | **Cluster C** | **Cluster D** | **Cluster E** | **Cluster F** | **Cluster G** | **Total** |
| --- | --- | --- | --- | --- | --- | --- | --- | --- | --- |
| **Before COVID-19 period**  **(N=5288)** | Sample size, n (%) | 1070 (52.9) | 443 (57.6) | 905 (68.8) | 474 (59.9) | 549 (50.4) | 617 (53.9) | 1230 (50.8) | 5288 (55.4) |
|  | Average MPR, % | 90.9 | 62.6 | 58.8 | 38.7 | 24.8 | 19.4 | 7.8 | 43.8 |
| **After COVID-19 period**  **(N=2569)** | Sample size, n (%) | 577 (28.5) | 195 (25.4) | 232 (17.6) | 173 (21.9) | 348 (31.9) | 315 (27.5) | 729 (30.1) | 2569 (26.9) |
|  | Average MPR, % | 92.7 | 63.7 | 61.6 | 40.4 | 21.8 | 18.7 | 7.1 | 41.2 |

GBTM, group-based trajectory modeling; MPR, medication possession ratio.

**e-Table 5. Summary of asthma-related ED visits, hospitalizations, and corticosteroid use before (March 1, 2020; n=6984) and after (n=4265) the COVID-19 period across seven GBTM clusters during 12-month follow-up**

|  | **Cluster A**  **Highest adherent** | **Cluster B**  **Late-stage stopper** | **Cluster C**  **Intermittent adherence** | **Cluster D**  **Half-way stopper** | **Cluster E**  **Fluctuated stopper** | **Cluster F**  **Delayed stopper** | **Cluster G**  **Lowest adherent** | **Total** |
| --- | --- | --- | --- | --- | --- | --- | --- | --- |
| **Asthma-related ED visits** | | | | | | | | |
| Patients with ≥1 event, n (%)  Pre-COVID  After COVID | 108 (7.5)  61 (6.4) | 48 (8.4)  28 (8.6) | 83 (7.7)  28 (6.8) | 59 (9.6)  37 (11.7) | 62 (8.4)  38 (7.0) | 95 (11.5)  51 (9.7) | 178 (10.5)  78 (6.6) | 633 (9.1)  321 (7.5) |
| Total events per patient during follow-up, mean (SD)  Pre-COVID  After COVID | 0.14 (0.67)  0.12 (0.93) | 0.20 (0.95)  0.12 (0.46) | 0.16 (0.76)  0.11 (0.54) | 0.23 (0.97)  0.27 (0.98) | 0.23 (1.23)  0.13 (0.63) | 0.22 (0.76)  0.17 (0.66) | 0.23 (0.99)  0.15 (0.91) | 0.20 (0.90)  0.15 (0.80) |
| Rate (events per 1000 patient days)  Pre-COVID  After COVID | 0.44  0.42 | 0.60  0.43 | 0.47  0.41 | 0.71  0.97 | 0.73  0.46 | 0.68  0.60 | 0.73  0.50 | 0.61  0.51 |
| Relative mean change before versus after COVID  beginning, % | –14.3 | –40.0 | –31.3 | 17.4 | –43.5 | –22.7 | –34.8 | –25.0 |
| **Asthma-related hospitalizations** | | | | | | | | |
| Patients with ≥1 event, n (%)  Pre-COVID  After COVID | 85 (5.9)  19 (2.0) | 40 (7.0)  9 (2.8) | 68 (6.3)  17 (4.2) | 53 (8.6)  14 (4.4) | 61 (8.2)  18 (3.3) | 76 (9.2)  19 (3.6) | 112 (6.6)  32 (2.7) | 495 (7.1)  128 (3.0) |
| Total events per patient during follow-up, mean (SD)  Pre-COVID  After COVID | 0.08 (0.39)  0.03 (0.21) | 0.09 (0.37)  0.03 (0.19) | 0.09 (0.39)  0.05 (0.26) | 0.17 (0.74)  0.06 (0.33) | 0.13 (0.85)  0.05 (0.29) | 0.14 (0.56)  0.05 (0.31) | 0.09 (0.45)  0.04 (0.32) | 0.11 (0.53)  0.04 (0.28) |
| Rate (events per 1000 patient days)  Pre-COVID  After COVID | 0.26  0.09 | 0.27  0.11 | 0.26  0.18 | 0.52  0.23 | 0.40  0.16 | 0.44  0.16 | 0.30  0.14 | 0.33  0.14 |
| Relative mean change before versus after COVID  beginning, % | –62.5 | –66.7 | –44.4 | –64.7 | –61.5 | –64.3 | –55.6 | –63.6 |
| **Oral or intravenous corticosteroid use** | | | | | | | | |
| Patients with ≥1 event, n (%)  Pre-COVID  After COVID | 494 (34.1)  241 (25.3) | 195 (34.0)  86 (26.4) | 395 (36.5)  111 (27.1) | 225 (36.4)  74 (23.3) | 240 (32.4)  133 (24.6) | 270 (32.6)  126 (23.9) | 540 (31.9)  300 (25.2) | 2359 (33.8)  1071 (25.1) |
| Total events per patient during follow-up, mean (SD)  Pre-COVID  After COVID | 1.09 (2.62)  0.73 (2.08) | 1.22 (2.62)  0.67 (1.47) | 1.19 (2.45)  0.64 (1.52) | 1.39 (3.31)  0.64 (1.62) | 1.22 (2.83)  0.71 (1.95) | 1.24 (3.37)  0.77 (2.13) | 1.22 (3.01)  0.69 (1.87) | 1.21 (2.88)  0.70 (1.89) |
| Rate (events per 1000 patient days)  Pre-COVID  After COVID | 3.47  2.47 | 3.74  2.36 | 3.51  2.32 | 4.31  2.30 | 3.79  2.40 | 3.89  2.66 | 3.88  2.33 | 3.75  2.41 |
| Relative mean change before versus after COVID  beginning, % | –33.0 | –45.1 | –46.2 | –54.0 | –41.8 | –37.9 | –43.4 | –42.1 |

ED, emergency department; GBTM, group-based trajectory modeling; SD, standard deviation.
